# Association Between Internet Use and Physical Health, Mental Health, and Subjective Health in Middle-aged and Older Adults: Nationally Representative Cross-sectional Survey in China

**DOI:** 10.2196/40956

**Published:** 2023-03-21

**Authors:** Wen Wen, Yaru Zhang, Wenjie Shi, Jiajia Li

**Affiliations:** 1 School of Public Health Shandong University Jinan China; 2 NHC Key Lab of Health Economics and Policy Research (Shandong University) Jinan China

**Keywords:** internet use, health status, middle-aged and older adults, China

## Abstract

**Background:**

Internet use is an important means of accessing health-related information. Identifying the associations between internet use and health outcomes could provide insight into strategies for improving public health among middle-aged and older adults (45 years and up).

**Objective:**

This study aimed to examine the relationship between internet use and health outcomes in middle-aged and older adults.

**Methods:**

Data were obtained from the 2018 China Health and Retirement Longitudinal Study. Physical, mental, and subjective health were assessed using the Activities of Daily Living (ADL) Scale, the 10-item Center for Epidemiologic Studies Depression Scale, and the 3-level Self-Rated Health Scale, respectively. The chi-square test and rank sum test were used to explore whether internet use was associated with health status. A multivariate logistic regression model was used to determine this association further after controlling for the confounding factors.

**Results:**

Overall, 13% (1752/13,474) of the participants used the internet. Regression analyses revealed that the prevalence of depression (odds ratio [OR] 0.59, 95% CI 0.52-0.68; *P*<.001), negative self-rated health (OR 0.68, 95% CI 0.61-0.76; *P*<.001), and difficulty with ADL (OR 0.48, 95% CI 0.39-0.60; *P*<.001) in the participating middle-aged and older adult was lower in those using the internet than nonusers. After controlling for confounding factors, internet use was found to be negatively associated with difficulty with ADL (urban: OR 0.44, 95% CI 0.32-0.61; *P*<.001 vs rural: OR 0.55, 95% CI 0.41-0.75; *P*<.001), depression (urban: OR 0.69, 95% CI 0.57-0.84; *P*<.001 vs rural: OR 0.52, 95% CI: 0.43-0.63; *P*<.001), and self-rated health status (urban: OR 0.70, 95% CI 0.61-0.81; *P*<.001 vs rural: OR 0.67, 95% CI 0.57-0.78; *P*<.001) among middle-aged and older adults in both urban and rural areas.

**Conclusions:**

Internet use had a positive effect on the physical and mental health of middle-aged and older adults who participated in this study. However, the internet usage rate remains low among older Chinese people. Therefore, the internet penetration rate should be a priority.

## Introduction

The global population is aging at an unprecedented rate. Between 2020 and 2030, the proportion of the global population aged 60 years and above is estimated to increase by 34% [1]. China is no exception, and 18.7% of its total population was aged 60 years and above in 2020 [2]. Health is undoubtedly the most prominent issue in the aging process [3]. As age increases, organ functions begin to deteriorate, making older individuals increasingly vulnerable to disease risks. Morbidity and mortality due to chronic diseases are mainly seen in older adults [4]. Additionally, longevity exacerbates the disease burden in the later stages of life [5]. Improving the health of older adults is an important challenge in the field of public health worldwide [6]. In China, inadequate access to health care services is significantly associated with higher rates of disability, cognitive impairment, and all-cause mortality among older adults [7]. However, within the context of an urban-rural dualistic social structure, there exists an inequality of opportunities in health care between urban and rural areas [8]. This is significant, as social participation was shown to be beneficial for the mental health of both urban and rural older adults [9]. Due to the combined effects of population aging and rural-urban labor migration, older adults who were left behind reported weaker family relationships and poorer mental health [10].

Additionally, various social factors can affect health [11]. The internet is an important tool that provides a wide range of channels for both doctors and patients to access health-related information [12]. Information gathered from the internet can influence patients’ preferences for doctors and hospitals [13]. Additionally, doctors can diagnose diseases using data collected remotely from patients [14]. Internet use has also been demonstrated to help reduce the risk of cognitive decline [15] and lower the prevalence of chronic diseases [16]. It also showed a correlation with self-rated health status or quality of life in older adults [17-19]. Moreover, internet use helps maintain kinship ties, enhances bonding between older adults and their children, and has a positive impact on subjective well-being [20]. Previous research has established that internet learning and usage may have beneficial effects on the aging brain [21]. Internet use helps remediate feelings of loneliness; therefore, older adults who use the internet are less likely to feel lonely or suffer from depression [22]. Health-related data based on internet search access can be used as a supplementary source for hospital monitoring systems. For example, health outcomes are predicted using Google’s flu prediction model [23], which can effectively provide benchmarks for disease prevention and enable the development of appropriate prevention and control policies.

However, fewer studies have presented different perspectives on internet usage and health among middle-aged and older adults. There are substantial variations in the information acquired from limited-income countries and high-income countries [24]. Nevertheless, some general problems are common among these countries. Older adults have low internet usage rates and are more at risk of digital exclusion [25-28]. As an information-vulnerable group, older adults are often physically fragile, cognitively limited [29], and lack basic information literacy [30-32]. They can rarely benefit from technological literacy and the ability to seek health-related information on the internet [33]. Thus, they are less engaged and less comfortable with using the internet [34]. Because older adults are vulnerable to the “digital divide,” the association between internet usage and health outcomes requires further research [16]. Although only a small proportion of middle-aged and older adults in China use the internet, this proportion is growing rapidly; therefore, we focused on this population group in our study. To explore the association between internet usage and health status according to the latest data from the 2018 China Health and Retirement Longitudinal Study (CHARLS), we searched previous studies with empirical analysis and evaluated the health outcomes comprehensively using a combination of subjective and objective indicators. This study also focused on comparing the internet-health association between residents of rural and urban areas. We intended to raise awareness of digital health among policy makers, health care workers, and the general public by assessing the robustness of the internet-health association.

## Methods

### Data Source

CHARLS began in 2011 [35], with participants followed up every 2 years. At present, CHARLS has 4 waves of data (2011, 2013, 2015, and 2018). Data were collected from Chinese citizens aged 45 and above from the 2018 CHARLS. The survey was extensive, covering 28 provinces, 150 county-level units, and 450 village-level units in China [35]. In this study, we excluded 168 individuals aged below 45 years and 4228 individuals with missing data, thus including 13,474 final valid samples.

### Ethical Considerations

Prior to the survey, each participant was given written informed consent specifying the purpose of this study. Ethical approval for all the CHARLS waves was granted by the institutional review board of Peking University (IRB00001052-11015). The survey was also anonymous, and the answers were protected by privacy law.

### Explained Variables

Health describes more than the mere integrity of the physical body [36]. It refers to a state of well-being in all aspects and needs to be measured using a comprehensive set of indicators. This study examined the relationship between internet use and the health status of middle-aged and older adults in 3 dimensions: physical health, mental health, and subjective self-rated health status. The main measure of physical health was conducted using the Activities of Daily Living (ADL) scale comprising the following six items: (1) dressing; (2) bathing or showering; (3) self-feeding; (4) getting into/out of bed; (5) using the toilet（including getting up and down); and (6) controlling urination and defecation (including using a catheter or a pouch by yourself). Each of the answers was categorized into four levels: (1) don’t have any difficulty, (2) have difficulty but can still do it, (3) have difficulty and need help, and (4) cannot do it. Participants who selected the “don’t have any difficulty” option were classified as having no difficulty, while those who selected the 3 remaining options were categorized as having difficulty [37,38]. Specifically, participants who had difficulty with any of the 6 items were recorded as having difficulty with ADL. No difficulty in any of the ADL items was scored as 0, and at least 1 difficulty was scored as 1 [37]. Mental health expressed as “depression” was measured using the 10-item Center for Epidemiologic Studies Depression Scale (CESD-10). The CESD-10, which was used to assess how the participants felt and behaved in the past week [39], comprises 2 positive and 8 negative questions. The negative issues are rated as 0 (symptoms for less than 1 day), 1 (symptoms for 1-2 days), 2 (symptoms for 3-4 days), and 3 (symptoms for 5-7 days), with the opposite rating for positive issues. A total score of 10 or higher indicates that the participant has depressive symptoms, and less than 10 indicates that the participant does not have depression [39]. Self-rated health status can be categorized as very good, good, fair, poor, and very poor; however, we classified it into 3 categories with a score of 1 for very good or good (positive), 2 for fair, and 3 for poor or very poor (negative) [37].

### Explanatory Variables

In this study, internet use was considered the core explanatory variable, with 1 representing internet access and 0 otherwise. After reviewing the literature, we organized the control variables according to the following demographic characteristics: sex (male=1, female=2), age (continuous variable), marital status (married=1, unmarried=0), education (below middle school=1, high school and vocational training=2, above high school=3), chronic diseases (yes=1, no=0), health insurance (yes=1, no=0), household expenditure per capita (continuous variable), place of residence (rural=1, urban=0), number of children (continuous variable), and interaction with friends (yes=1, no=0). The household expenditure per capita was added to “1” and then logged. For marital status, “unmarried” included separated, divorced, widowed, and never married options.

### Data Analysis

Stata version 14.0 software (StataCorp), was used for all statistical analyses; *P* values <0.05 were considered statistically significant (2-sided). Means and SDs were used to describe the continuous data, and frequencies and percentages were used to describe the categorical data. Univariate analysis was used to test the association between internet use and health outcomes. The chi-square test was used for categorical variables and the rank-sum test for continuous variables that did not show normal distribution. The ADL and CESD-10 scores were analyzed as dependent and dichotomous variables. Self-rated health status was evaluated as a trichotomous variable. Therefore, we used a binary logistic regression model for ADL and depression and the ologit model for self-rated health to investigate the odds ratios (ORs) and 95% CIs. Further analysis was performed using grouped regression analysis for the urban and rural subgroups.

## Results

### Sample Description and Univariate Analysis Results

As shown in Tables 1-3, the sample consisted of 13,474 individuals, among which 6526 (48.43%) were male and 6948 (51.57%) were female. The average age was 61.5 years. Most of the respondents (11,783/13,474, 87.45%) had a low educational level, roughly one-tenth (1447/13,474, 10.74%) had high school and vocational training, and only a few (244/13,474, 1.81%) were highly educated. Most of the sample consisted of internet nonusers (11,722/13,474, 87% vs 1752/13,474, 13%). Among the participants who had difficulties with any item of the ADL, 98 (4.07%) used the internet and 2307 (95.93%) did not use the internet. Among those with depressive symptoms, 4182 (92.52%) did not use the internet. Of those who had a positive self-rated health status, 622 (19.35%) used the internet, and of those who had a negative self-rated health status, 226 (6.29%) used the internet. In comparison to healthy participants, those experiencing difficulties in ADL, depressive symptoms, and negative subjective self-rated health were more likely not to use the internet (*P*<.001) and be female (*P*<.001), older (*P*<.001), educated below the middle school level (*P*<.001), and more likely to suffer from chronic illness (*P*<.001). Regarding social and health-related factors, older adults with physical and mental health struggles or negative subjective self-rated health were more likely to have lower per capita household expenditures (*P*<.001), live in rural areas (*P*<.001), and have more children (*P*<.001) and fewer friends (*P*<.001).

**Table 1:** Description and univariate analysis results for study participants (N=13,474)^a,b,c^.

Variable		All	Unrestricted	Restricted	*P* value
**Internet use, n (%)**					<.001
	Yes	1752 (13)	1654 (14.94)	98 (4.07)	
	No	11,722 (87)	9415 (85.06)	2307 (95.93)	
**Sex, n (%)**					<.001
	Male	6526 (48.43)	5619 (50.76)	907 (37.71)	
	Female	6948 (51.57)	5450 (49.24)	1498 (62.29)	
Age (years), mean (SD)		61.50 (9.30)	60.54 (9.01)	65.93 (9.32)	<.001
**Marital status, n (%)**					<.001
	Married	11,737 (87.11)	9844 (88.93)	1893 (78.71)	
	Unmarried	1737 (12.89)	1225 (11.07)	512 (21.29)	
**Education, n (%)**					<.001
	Below middle school	11,783 (87.45)	9521 (86.01)	2262 (94.05)	
	High school and vocational training	1447 (10.74)	1315 (11.88)	132 (5.49)	
	Above high school	244 (1.81)	233 (2.10)	11 (0.46)	
**Chronic disease, n (%)**					<.001
	Yes	5915 (43.90)	4493 (40.59)	1422 (59.13)	
	No	7559 (56.10)	6576 (59.41)	983 (40.87)	
**Health insurance, n (%)**					<.001
	Yes	11,127 (82.58)	9042 (81.69)	2085 (86.69)	
	No	2347 (17.42)	2027 (18.31)	320 (13.31)	
Household expenditure per capita, mean (SD)		9.34 (0.97)	9.37 (0.96)	9.19 (1.04)	<.001
**Place of residence, n (%)**					<.001
	Urban	5038 (37.39)	4328 (39.10)	710 (29.52)	
	Rural	8436 (62.61)	6741 (60.90)	1695 (70.48)	
Number of children, mean (SD)		2.54 (1.30)	2.43 (1.23)	3.04 (1.45)	<.001
**Interacted with friends, n (%)**					<.001
	Yes	4628 (34.35)	3900 (35.23)	728 (30.27)	
	No	8846 (65.65)	7169 (64.77)	1677 (69.73)	

^a^Regarding the statistical description of variables, we used mean and SD for continuous variables and frequency (n) and percentage for categorical variables.

^b^Physical health was classified into 2 types: restricted (having difficulty in any item of activities of daily living [ADL]) and unrestricted (having no difficulty with ADL).

^c^The internet usage rate was 20.98% (1057/5038) in urban and 8.24% (695/8436) in rural areas.

**Table 2:** Description and univariate analysis results for study participants (N=13,474)^a^.

Variable		All	No depression	Depression	*P* value	
**Internet use, n (%)**					<.001	
	Yes	1752 (13)	1414 (15.79)	338 (7.48)		
	No	11,722 (87)	7540 (84.21)	4182 (92.52)		
**Sex, n (%)**					<.001	
	Male	6526 (48.43)	4791 (53.51)	1735 (38.38)		
	Female	6948 (51.57)	4163 (46.49)	2785 (61.62)		
Age (years), mean (SD)		61.50 (9.30)	61.28 (9.33)	61.95 (9.23)	<.001	
**Marital status, n (%)**					<.001	
	Married	11,737 (87.11)	7971 (89.02)	3766 (83.32)		
	Unmarried	1737 (12.89)	983 (10.98)	754 (16.68)		
**Education, n (%)**					<.001	
	Below middle school	11,783 (87.45)	7595 (84.82)	4188 (92.65)		
	High school and vocational training	1447 (10.74)	1147 (12.81)	300 (6.64)		
	Above high school	244 (1.81)	212 (2.37)	32 (0.71)		
**Chronic disease, n (%)**					<.001	
	Yes	5915 (43.90)	3534 (39.47)	2381 (52.68)		
	No	7559 (56.10)	5420 (60.53)	2139 (47.32)		
**Health insurance, n (%)**					<.001	
	Yes	11,127 (82.58)	7148 (79.83)	3979 (88.03)		
	No	2347 (17.42)	1806 (20.17)	541 (11.97)		
Household expenditure per capita, mean (SD)		9.34 (0.97)	9.38 (0.96)	9.25 (0.99)	<.001	
**Place of residence, n (%)**					<.001	
	Urban	5038 (37.39)	3687 (41.18)	1351 (29.89)		
	Rural	8436 (62.61)	5267 (58.82)	3169 (70.11)		
Number of children, mean (SD)		2.54 (1.30)	2.47 (1.28)	2.68 (1.31)	<.001	
**Interacted with friends, n (%)**					<.001	
	Yes	4628 (34.35)	3156 (35.25)	1472 (32.57)		
	No	8846 (65.65)	5798 (64.75)	3048 (67.43)		

^a^Regarding the statistical description of variables, we used mean and SD for continuous variables and frequency (n) and percentage for categorical variables.

**Table 3:** Description and univariate analysis results for study participants (N=13,474)^a,b^.

Variable				All		Positive		General		Negative		*P* value
**Internet use, n (%)**												<.001
		Yes		1752 (13)		622 (19.35)		904 (13.56)		226 (6.29)		
		No		11,722 (87)		2592 (80.65)		5765 (86.44)		3365 (93.71)		
**Sex, n (%)**												<.001
		Male		6526 (48.43)		1741 (54.17)		3232 (48.46)		1553 (43.25)		
		Female		6948 (51.57)		1473 (45.83)		3437 (51.54)		2038 (56.75)		
Age (years), mean (SD)				61.50 (9.30)		60.33 (9.13)		61.09 (9.24)		63.32 (9.31)		<.001
**Marital status, n (%)**												<.001
		Married		11,737 (87.11)		2841 (88.39)		5905 (88.54)		2991 (83.29)		
		Unmarried		1737 (12.89)		373 (11.61)		764 (11.46)		600 (16.71)		
**Education, n (%)**												<.001
	Below middle school		11,783 (87.45)		2628 (81.77)		5822 (87.30)		3333 (92.82)			
	High school and vocational training		1447 (10.74)		479 (14.90)		734 (11.01)		234 (6.52)			
	Above high school		244 (1.81)		107 (3.33)		113 (1.69)		24 (0.67)			
**Chronic disease, n (%)**												<.001
	Yes		5915 (43.90)		891 (27.72)		2784 (41.75)		2240 (62.38)			
	No		7559 (56.10)		2323 (72.28)		3885 (58.25)		1351 (37.62)			
**Health insurance, n (%)**												<.001
	Yes		11,127 (82.58)		2515 (78.25)		5456 (81.81)		3156 (87.89)			
	No		2347 (17.42)		699 (21.75)		1213 (18.19)		435 (12.11)			
Household expenditure per capita, mean (SD)				9.34 (0.97)		9.41 (1)		9.35 (0.96)		9.24 (0.97)		<.001
**Place of residence, n (%)**												<.001
	Urban		5038 (37.39)		1383 (43.03)		2605 (39.06)		1050 (29.24)			
	Rural		8436 (62.61)		1831 (56.97)		4064 (60.94)		2541 (70.76)			
Number of children, mean (SD)				2.54 (1.30)		2.38 (1.21)		2.48 (1.27)		2.80 (1.38)		<.001
**Interacted with friends, n (%)**												<.001
	Yes		4628 (34.35)		1249 (38.86)		2324 (34.85)		1055 (29.38)			
	No		8846 (65.65)		1965 (61.14)		4345 (65.15)		2536 (70.62)			

^a^Regarding the statistical description of variables, we used mean and SD for continuous variables and frequency (n) and percentage for categorical variables.

^b^Self-reported health was categorized into 3 types: positive (very good or good), fair, and negative (poor or very poor).

### Regression Analysis Results

Table 4 summarizes the regression analysis results and shows the relationship between internet use and each health dimension. Internet use was shown to have a significant negative association with ADL performance, depression, and self-rated health status. The results revealed that individuals who used the internet were 0.48 times more likely to have difficulties with ADL than those who did not use the internet (OR 0.48, 95% CI 0.39-0.60; *P*<.001). In addition, older adults who used the internet had 0.59 times the odds of depression than nonusers (OR 0.59, 95% CI 0.52-0.68; *P*<.001). Moreover, participants who used the internet had significantly lower odds for negative self-rated health status than nonusers (OR 0.68, 95% CI 0.61-0.76; *P*<.001). The prevalence of restricted ADL, depression, and negative self-rated health status decreased with increased internet use, indicating that it is a protective factor for health.

**Table 4:** Regression results for ADL^a^, depression^b^, and subjective self-rated health^c^ status among study participants (N=13,474).

Variable		ADL		Depression			Self-rated health	
		OR^d^ (95% CI)	*P* value	OR (95% CI)	*P* value	OR (95% CI)		*P* value
**Internet use**								
	Yes (ref: no)	0.48 (0.39-0.60)	<.001	0.59 (0.52-0.68)	<.001	0.68 (0.61-0.76)		<.001
**Sex**								
	Female (ref: male)	1.71 (1.55-1.88)	<.001	1.72 (1.60-1.86)	<.001	1.30 (1.22-1.39)		<.001
Age		1.05 (1.04-1.06)	<.001	0.99 (0.99-1.00)	.075	1.01 (1-1.02)		<.001
**Marital status**								
	Married (ref: unmarried)	0.79 (0.69-0.90)	<.001	0.68 (0.61-0.77)	<.001	0.91 (0.82-1.01)		.078
**Education**								
	High school and vocational training (ref: below middle school)	0.77 (0.63-0.94)	.012	0.73 (0.63-0.84)	<.001	0.78 (0.70-0.87)		<.001
	Above high school (ref: below middle school)	0.43 (0.23-0.80)	.007	0.61 (0.41-0.90)	.013	0.63 (0.48-0.81)		<.001
**Chronic diseases**								
	Yes (ref: no)	2.12 (1.93-2.33)	<.001	1.74 (1.61-1.87)	<.001	2.68 (2.51-2.87)		<.001
**Health insurance**								
	Yes (ref: no)	1.11 (0.95-1.29)	.181	1.26 (1.12-1.42)	<.001	1.18 (1.07-1.30)		.001
Household expenditure per capita		0.97 (0.93-1.02)	.18	0.97 (0.93-1.01)	.139	0.99 (0.96-1.03)		.755
**Place of residence**								
	Rural (ref: urban)	1.33 (1.20-1.49)	<.001	1.40 (1.29-1.52)	<.001	1.30 (1.21-1.40)		<.001
Number of children		1.10 (1.06-1.15)	<.001	1.05 (1.01-1.08)	.008	1.05 (1.02-1.08)		.001
**Interacted with friends**								
	Yes (ref: No)	0.88 (0.80-0.98)	.015	0.94 (0.87-1.02)	.149	0.81 (0.76-0.87)		<.001

^a^ADL: activities of daily living. Reporting difficulty with ADL (ref: no).

^b^Depression (ref: no).

^c^Self-rated health (ref: reporting positive self-reported health).

^d^OR: odds ratio.

The logistic regression was also conducted to identify an association between several factors and health (Table 4). The odds of restricted ADL (OR 1.71, 95% CI 1.55-1.88; *P*<.001), depression (OR 1.72, 95% CI 1.60-1.86; *P*<.001), and negative self-rated health status (OR 1.30, 95% CI 1.22-1.39; *P*<.001) were greater in females than in males. A higher educational level was significantly associated with ADL performance (OR 0.43, 95% CI 0.23-0.80; *P*=.007), depression (OR 0.61, 95% CI 0.41-0.90; *P*=.013), and self-rated health status (OR 0.63, 95% CI 0.48-0.81; *P*<.001). Furthermore, having a chronic illness led to significantly higher odds of ADL difficulties (OR 2.12, 95% CI 1.93-2.33; *P*<.001), depression (OR 1.74, 95% CI 1.61-1.87; *P*<.001), and negative subjective self-rated health (OR 2.68, 95% CI 2.51-2.87; *P*<.001). The odds of restricted ADL (OR 1.33, 95% CI 1.20-1.49; *P*<.001), depression (OR 1.40, 95% CI 1.29-1.52; *P*<.001), and negative self-rated health status (OR 1.30, 95% CI 1.21-1.40; *P*<.001) were significantly higher in those living in rural areas than in those living in urban areas.

Similarly, having more children was positively associated with ADL performance (OR 1.10, 95% CI 1.06-1.15; *P*<.001), depressive conditions (OR 1.05, 95% CI 1.01-1.08; *P*=.008), and self-rated health status (OR 1.05, 95% CI 1.02-1.08; *P*=.001). Finally, respondents who interacted with friends in the past month showed lower odds with ADL performance (OR 0.88, 95% CI 0.80-0.98; *P*=.015) and negative self-rated health status (OR 0.81, 95% CI 0.76-0.87; *P*<.001), but there was no significant association with depression. There were no significant associations between household expenditure per capita and overall health status. As shown in Table 5, for the rural and urban subgroups, it is concerning that individuals who use the internet showed a lower chance of ADL performance (urban: OR 0.44, 95% CI 0.32-0.61; *P*<.001 vs rural: OR 0.55, 95% CI 0.41-0.75; *P*<.001), depression (urban: OR 0.69, 95% CI 0.57-0.84; *P*<.001 vs rural: OR 0.52, 95% CI: 0.43-0.63; *P*<.001), and negative self-rated health status (urban: OR 0.70, 95% CI 0.61-0.81; *P*<.001 vs rural: OR 0.67, 95% CI 0.57-0.78; *P*<.001) than nonusers.

**Table 5:** Results for urban versus rural subgroups (N=13,474).

Variable		ADL^a^						Depression^b^						Self-rated health^c^				
		Urban			Rural			Urban			Rural			Urban		Rural		
		OR^d^ (95 % CI)	*P* value	OR (95% CI)		*P* value	OR (95 %CI)		*P* value	OR (95% CI)		*P* value	OR (95% CI)		*P* value		OR (95% CI)	*P* value
**Internet use**																		
	Yes (ref: no)	0.44 (0.32-0.61)	<.001	0.55 (0.41-0.75)		<.001	0.69 (0.57-0.84)		<.001	0.52 (0.43-0.63)		<.001	0.70 (0.61-0.81)		<.001		0.67 (0.57-0.78)	<.001
**Sex**																		
	Female (ref: male)	1.40 (1.18-1.67)	<.001	1.89 (1.68-2.13)		<.001	1.60 (1.40-1.83)		<.001	1.79 (1.63-1.97)		<.001	1.24 (1.11-1.38)		<.001		1.34 (1.23-1.46)	<.001
Age		1.05 (1.04-1.06)	<.001	1.05 (1.04-1.06)		<.001	0.99 (0.98-0.99)		.003	1.00 (0.99-1.01)		.920	1.01 (1-1.02)		.002		1.02 (1.01-1.02)	<.001
**Marital status**																		
	Married (ref: unmarried)	0.91 (0.72-1.16)	.454	0.74 (0.63-0.87)		<.001	0.65 (0.54-0.80)		<.001	0.71 (0.62-0.81)		<.001	0.84 (0.70-0.99)		.044		0.96 (0.84-1.09)	.486
**Education**																		
	High school and vocational training (ref: below middle school)	0.89 (0.67-1.18)	.428	0.72 (0.54-0.96)		.024	0.82 (0.67-1.01)		.061	0.65 (0.53-0.80)		<.001	0.77 (0.66-0.90)		.001		0.79 (0.67-0.93)	.004
	Above high school (ref: below middle school)	0.42 (0.20-0.88)	.033	0.73 (0.27-1.96)		.530	0.60 (0.39-0.93)		.024	0.79 (0.31-2.03)		.630	0.64 (0.48-0.85)		.002		0.46 (0.22-0.96)	.038
**Chronic diseases**																		
	Yes (ref: no)	2.01 (1.70-2.39)	<.001	2.18 (1.95-2.45)		<.001	1.64 (1.44-1.86)		<.001	1.79 (1.63-1.96)		<.001	2.43 (2.18-2.72)		<.001		2.83 (2.60-3.09)	<.001
**Medical insurance**																		
	Yes (ref: no)	1.33 (1.07-1.65)	.009	0.88 (0.71-1.08)		.224	1.26 (1.07-1.49)		.005	1.19 (0.99-1.43)		.063	1.12 (0.98-1.28)		.097		1.22 (1.05-1.43)	.012
Household expenditure per capita		0.99 (0.90-1.09)	.847	0.97 (0.92-1.03)		.375	0.99 (0.92-1.08)		.978	0.97 (0.92-1.01)		.147	1.01 (0.95-1.08)		.714		0.99 (0.95-1.03)	.578
Number of children		1.16 (1.09-1.24)	<.001	1.07 (1.02-1.12)		.009	1.18 (1.11-1.25)		<.001	0.99 (0.95-1.03)		.499	1.12 (1.07-1.18)		<.001		1.02 (0.98-1.06)	0.332
**Interacted with friends**																		
	Yes (ref: no)	0.82 (0.69-0.99)	.040	0.91 (0.81-1.03)		.126	0.78 (0.68-0.90)		.001	1.04 (0.94-1.14)		.478	0.75 (0.67-0.84)		<.001		0.84 (0.77-0.92)	<.001

^a^ADL: activities of daily living. Reporting difficulty with ADL (ref: no).

^b^Depression (ref: no).

^c^Self-rated health (ref: reporting positive self-reported health).

^d^OR: odds ratio.

## Discussion

### Principal Findings

Internet use has a significant positive association with the health of middle-aged and older adults. This could be attributed to the vast resources of the internet that have laid the foundation for guiding people toward a healthy lifestyle. With the improvement in living standards and increase in age, people pay more attention to their own health and are more inclined to search for health-related information when required. There are abundant health resources on the internet, and people can search for relevant information in real time using their cell phones or other digital devices [40]. Moreover, internet usage among older adults has also been associated with better financial and health care decisions [41]. People who use the internet are likely to have more wealth and therefore are more likely to be physically and mentally healthier in the first place [42]. The internet has enabled people to acquire health-related knowledge and gradually develop healthy lifestyle habits, improving their overall health.

However, China started utilizing the internet later than Western countries [43]. Therefore, older adults may have technical barriers to internet use or lack the technical knowledge and skills to use these technologies [44]. The internet usage rate of the older adults in our study was 13% (1752/13,474), which is lower than the internet usage rate of older adults in other countries (between 42.6% and 82%) [45-47]. People aged 45 years and up face “digital divide” barriers due to the lack of necessary internet equipment or skills [38,48]. According to the data in the China Statistical Report on Internet Development in 2018, Chinese netizens are mainly aged between 10 and 39 years [49]; thus, middle-aged and older adults are digitally marginalized. In June 2018, the internet penetration rate was only 58% in China [50]. In contrast, digital development in Europe was more mature; in Iceland, it exceeded 95% in June 2016 [51].

Middle-aged and older adults with restricted mobility and financial burdens are faced not only with the daunting task of maintaining a normal life but also with enormous barriers to social interaction, which makes them prone to depression [52]. The internet can help reduce reliance on formal care systems [53] and build a convenient bridge of communication for them. Especially in rural areas, a large number of empty nesters can keep in touch with their children through the internet. Furthermore, the internet can provide a wealth of entertainment options and may be a potential mechanism to alleviate loneliness in older adults [54]. There is no denying that there is also an interaction effect between internet use and social isolation on depression. However, the negative effects of social isolation are stronger for older adults who use the internet less often [55]. Internet technology can also be used for disease diagnosis, screening, and assisting patients with treatment and care [56]. Moreover, middle-aged and older adults with physical impairments can meet their daily needs through online shopping and home-care appointments.

Self-rated health is an individual’s subjective evaluation and comprehensive assessment based on their actual health status. Related studies have shown that pain is associated with self-rated health [57]. Moreover, people with depressive symptoms and high stresses in life are less likely to have positive attitudes toward their health [57]. Self-rated health not only reflects an individual’s perception and evaluation of preexisting illnesses but also the symptoms of illness that have not been diagnosed yet [58]. Internet use is an important predictor of self-rated health [38]. Using the internet requires people to have the ability to operate complex technology. Older adults with a greater capacity to use smartphones have better self-rated health [59]. Notably, low-income individuals may be reluctant to use the internet and may not benefit from it because they have limited resources (eg, education or health resources) [60]. Internet users from affluent backgrounds with satisfactory living standards have better health knowledge and resources; therefore, they may have a better understanding of their health.

Influenced by the urban-rural dual structure, there is a large gap in the internet usage rate between rural and urban areas (8.24% vs 20.98%, respectively) in this study. Influential factors include physical geography, regional development policies, and misallocation of medical resources, which affect various health outcomes [37]. Additionally, the results of this study showed that internet use was associated with good health in both rural and urban dwellers. This may be because the internet compensates for geographical differences and social isolation [61], enabling older adults to reshape social networks and increase their social participation and better adapt to society, thereby promoting health [62,63].

Our findings revealed that female participants had poorer health than male participants. Generally, with respect to individual biological factors, women suffer greater morbidity, particularly late in life, and have worse self-rated health [64,65]. As people grow older, the incidence of chronic diseases increases, resulting in a heavy disease burden that affects their quality of life [66,67]. Regarding social support, marriage seems to have a positive impact on health. Spousal relationships are more influential than others, and high-quality marriages are associated with health benefits [68]. Social activities improve communication, stimulate cognitive functioning, provide emotional support [69], and positively affect the health of middle-aged and older adults [70].

The more children in a family, the greater the expenditure. According to the resource dilution theory, this is detrimental to the health of individuals to have limited access to resources [71]. In terms of socioeconomic status, education can improve health through healthy lifestyles, increased income, and improved subjective well-being [72]. Moreover, socioeconomic status positively affects health [73], and internet usage is associated with better financial and health care decision-making among older adults [41]. The association between insurance and health outcomes in our study is slightly different from that in previous studies [58], which could be attributed to the “adverse selection” of insurants with illnesses [74] or the limited role of socioeconomic status in the later stages of life [75]. This complicated correlation with the health of older adults warrants further research [76].

In addition, internet use may have a greater impact on the middle-aged population than on the older adult population. It may be that middle-aged people are more receptive to and understanding of information and are therefore better able to use the internet to improve their health [77].

### Limitations

This study has some limitations. Due to the large number of missing values removed for the main variables “depression” and “self-rated health” and to keep the sample size uniform, 31.3% (4228/13,474) of individuals from the 2018 CHARLS were excluded for missing data. Therefore, the representativeness may be limited due to the reduction of the sample size for analysis. The potential bias caused by sample selection may underestimate the association between internet use and health outcomes in middle-aged and older populations.

This study has further limitations. First, we aimed to incorporate more factors that may influence internet use and health. However, other variables may affect health and depression that were not included. Second, this was a cross-sectional study, and we could not explore the causal relationship between internet use and health. Third, it did not include the pathways by which internet use affects health. In the future, we plan to conduct further research through mechanistic exploration and heterogeneity analyses.

### Conclusions

This study examined the association between internet use and the physical, mental, and subjective self-rated health status among middle-aged and older adults and found that internet use has a positive effect on health status. The internet, a powerfully open and inclusive tool, is an important milestone in the development of human society. However, the massive usage of internet technology has largely amplified the age-related digital divide [78]. Nowadays, internet technology is widely used to support health care. However, older adults are often limited by their level of education, internet access, or confidence in using the internet, but they are also more likely to be the group most in need of medical support [79,80]. Particularly in rural areas, older adults are unfamiliar with internet use. Helping middle-aged and older adults adapt to the internet era should be a top priority.

First, future internet use should consider the accessibility of tools and develop access to appropriate programs for older adults. The government, communities, and enterprises should pay attention to the health of middle-aged and older adults, strengthen cooperation, and work together to promote aging-friendly internet applications. Second, they should guide middle-aged and older adults who are willing to use the internet to become familiar with it. On the one hand, the confidence of middle-aged and older adults in using the internet can be enhanced by offering training courses on cell phones and computers in the community. Another method could be to have younger people volunteer to go and teach or help older people in their homes, which would equally have benefits.

## Abbreviations

ADL: activities of daily living
CESD-10: 10-item Center for Epidemiologic Studies Depression Scale
CHARLS: China Health and Retirement Longitudinal Study
OR: odds ratio

## Data Availability

The data sets generated and/or analyzed during this study are available from the China Health and Retirement Longitudinal Study (CHARLS) data repository.
